# Treatment strategies for clear cell renal cell carcinoma: Past, present and future

**DOI:** 10.3389/fonc.2023.1133832

**Published:** 2023-03-21

**Authors:** Junwei Yang, Kuansong Wang, Zhichun Yang

**Affiliations:** ^1^ Xiangya School of Pharmaceutical Sciences, Central South University, Changsha, Hunan, China; ^2^ Department of Pathology, Xiangya Hospital, Central South University, Changsha, Hunan, China; ^3^ Department of Pathology, School of Basic Medical Science, Central South University, Changsha, Hunan, China; ^4^ National Clinical Research Center for Geriatric Disorders, Xiangya Hospital, Central South University, Changsha, China

**Keywords:** clear cell renal cell carcinoma, cytokine, tyrosine kinase inhibitor, immune checkpoint inhibitor, obesity paradox, renal cancer stem cells, immunological substance modification

## Abstract

Clear cell renal cell carcinoma (ccRCC) is the most prevalent histological subtype of kidney cancer, which is prone to metastasis, recurrence, and resistance to radiotherapy and chemotherapy. The burden it places on human health due to its refractory nature and rising incidence rate is substantial. Researchers have recently determined the ccRCC risk factors and optimized the clinical therapy based on the disease’s underlying molecular mechanisms. In this paper, we review the established clinical therapies and novel potential therapeutic approaches for ccRCC, and we support the importance of investigating novel therapeutic options in the context of combining established therapies as a research hotspot, with the goal of providing diversified therapeutic options that promise to address the issue of drug resistance, with a view to the early realization of precision medicine and individualized treatment.

## 1 Introduction

Kidney cancer is one of the most common malignancies of the urinary system. According to statistics, the number of new cases of kidney cancer in the world exceeded 430,000, and the number of new deaths was approximately 180,000 in 2020 (1). The American Cancer Society forecasts the top 10 cancers with the highest number of new cases in the United States in 2022. Kidney cancer ranks sixth among men and ninth among women (2). Renal cell carcinoma (RCC) accounts for more than 90% of kidney cancer. It originates from the renal tubular epithelium and has three common pathological types: clear cell RCC (ccRCC), papillary RCC, and chromophobe RCC (3). Of these, the most frequent is ccRCC, accounting for 70-75% (4).

**Figure 1:**
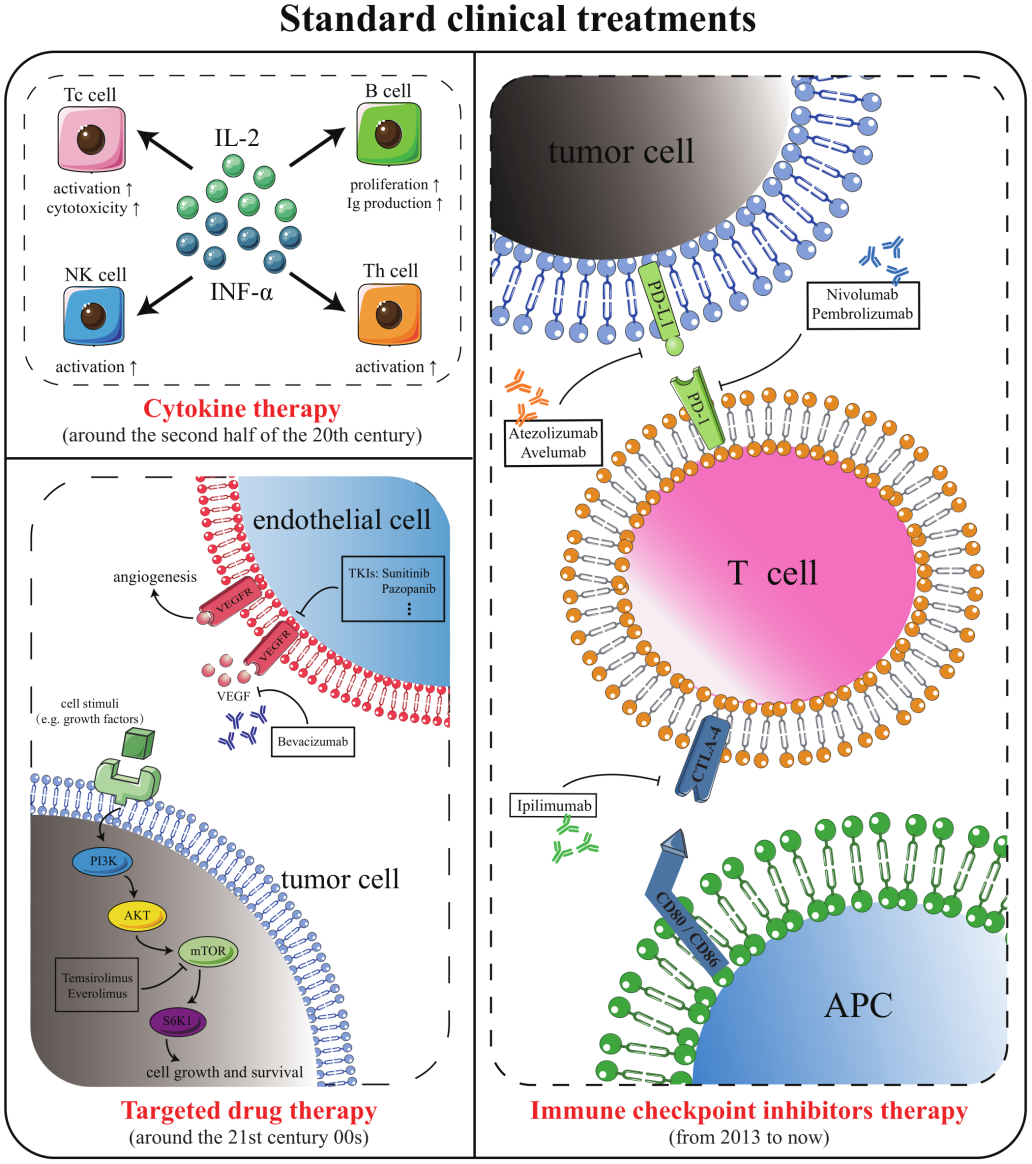
Standard methods for the treatment of mccRCC. Cytokine therapy, targeted drug therapy, and ICI therapy are the standard mccRCC treatment options that have emerged successively in clinical history.

Cytokines, targeted medications, and immune checkpoint inhibitors (ICI) have successively become the standard clinical options for metastatic ccRCC (mccRCC) (5, 6)(**Figure 1**). Although the survival advantages of these medications for patients with ccRCC are widely known, single-class drug therapy is vulnerable to drug resistance. Scholars are keen to combine standard clinical therapies to expand the options for treating medication resistance (**Table 1**). However, merely merging existing classical therapies does not address every issue. Effective interventions need to be developed for patients who are not sensitive to existing therapies, do not respond durably or fail treatment (3). Therefore, research into new prospective therapy techniques is still crucial in the field of ccRCC.

**Table 1:** Combination strategies of classical therapies on clinical trials in the last three years.

Combined treatment type	Specific combined treatment modalities	Phase	Identifier
Surgery (or ablation) - ICI	Nephrectomy - Pembrolizumab	Phase III	NCT03142334
	Nephrectomy - Atezolizumab	Phase III	NCT03024996
	Nephrectomy - Nivolumab	Phase III	NCT03055013
	Cryoablation - Tremelimumab	Phase I	NCT02626130
Targeted drug - Targeted drug	Lenvatinib - Everolimus	Phase III	NCT02811861
	Vorolanib - Everolimus	Phase III	NCT03095040
	Sunitinib - Erlotinib	Phase II	NCT00425386
Targeted drug - ICI	Lenvatinib - Pembrolizumab	Phase III	NCT02811861
	Axitinib - Pembrolizumab	Phase III	NCT02853331
	Axitinib - Avelumab	Phase III	NCT02684006
	Bevacizumab - Atezolizumab	Phase III	NCT02420821
	Cabozantinib - Nivolumab	Phase III	NCT03141177
	Cabozantinib - Atezolizumab	Phase Ib	NCT03170960
	Sitravatinib - Nivolumab	Phase I/II	NCT03680521
ICI-ICI	Nivolumab - Ipilimumab	Phase III	NCT02231749
Targeted drug - ICI - ICI	Cabozantinib - Nivolumab - Ipilimumab	Phase III	NCT03141177

In this review, we summarize the existing clinical treatments for ccRCC and discuss potential strategies for the future treatment of ccRCC. In addition, we also put forward our views and explanations on the necessity of cytoreductive nephrectomy (CN) for metastatic patients, the paradox of obesity, and the feasibility of tumor “slimming”.

## 2 Classical clinical treatments

### 2.1 Surgical operation and tumor ablation

Surgical resection is the recommended course of treatment for localized ccRCC (7). The commonly used nephrectomy modalities are radical nephrectomy (RN) and nephron-sparing surgery (NSS). RN means removing the entire kidney, perinephric adipose tissue (PAT), and Gerota’s fascia, where the lesion locates. It was once thought to be the only cure for localized renal cancer. However, RN leads to acute loss of nephron, sharply increases the load of the contralateral kidney, and can easily trigger acute postoperative kidney injury (8). NSS only removes part of the diseased kidney tissue, preserving as much of the ipsilateral normal kidney unit as possible. Nevertheless, NSS is a long procedure and is prone to common complications such as hematuria, perirenal hematoma, and urinary fistula (3, 9). As long as it is technically feasible, the guidelines recommend prioritizing NSS for patients with T1 and T2 (10).

The treatment modality to remove the primary tumor despite the metastasis has already occurred is known as CN (11). CN has long been considered as the standard of care for most patients with mccRCC. However, a recent clinical trial stated that sunitinib combined with CN was not superior to sunitinib alone and questioned CN as a form of overtreatment (12). Conversely, another study reported that the implementation of CN is beneficial for the survival of some patients with a giant primary tumor, fewer metastatic lesions, and better physical condition (12, 13). CN plays a synergistic role in immunotherapy by erasing the primary tumor and thus removing cytokines and proteins that inhibit the immune response (14). The controversial issue of CN prompts us that with advancing and evolving in medical techniques, the necessity of conventional surgical treatment for mccRCC patients should be evaluated properly. Rather than blindly denying the role of CN, we should accurately define the patients who can benefit from CN and recommend time of surgery for them through scientific means such as risk scoring (15).

Surgical procedures are less suitable for elderly patients with poor physical status, patients with bilateral tumors in the kidneys, and patients with only a single kidney remaining (16). The advent of tumor ablation has brought an effective alternative for such patients. Tumor ablation is a local tumor control method, which ablates the tumor and healthy tissues beside the tumor by placing a probe in the central area of the tumor under the guidance of imaging (17). Compared with traditional surgery, tumor ablation has the advantages of being minimally invasive, having a short treatment time, and being able to carry out repeatedly. Currently, the commonly used clinical ablation techniques are radiofrequency ablation (RFA), microwave ablation (MWA) and cryoablation (CA) (18). RFA and MWA are thermal ablations that use high temperatures to cause coagulation necrosis of cells (19). CA uses argon to cool the probe to −160 °C and below rapidly, then helium to slowly re-warm the target tumor to 20-40 °C, and the cooling and re-warming cycles repeat (16). This cold-hot alternating freeze-thaw cycle can trigger cellular damage, vascular damage, apoptosis, and immune effects in tumors (16, 20). Compared to thermal ablation, CA is less unpleasant, has distinct ablation boundaries, and is safer (21). However, the cooling capacity of CA is positively correlated with the diameter of the probe, which limits its ablative capacity (16). Moreover, CA is expensive and time-consuming compared to thermal ablation. Available studies have shown that for tumors ≤4 cm in diameter, the effectiveness of MWA and RFA treatment is indistinguishable, and thermal ablation is slightly more effective than CA (22, 23).

### 2.2 Cytokine therapy

For individuals with metastases, systemic therapy is not always necessary, but should be considered. Chemotherapy is one of the most well-known systemic treatments, but mccRCC is not sensitive to chemotherapy. The spontaneous tumor regression in a minimal number of mccRCC patients has focused attention on the anti-tumor response of the immune system. Cytokines, soluble proteins released by cells with the ability to modulate the immune system, were the first anti-cancer immune substances to receive attention, ushering in the era of prior immunotherapy (24). Studies have shown that the median overall survival (OS) of low-, intermediate-, and high-risk patients who received cytokine therapy was 27, 12, and 6 months, while the median OS of these three categories of patients who received chemotherapy was 15, 7, and 3 months, respectively (25). This suggests that cytokine therapy is slightly superior to chemotherapy in the treatment of mccRCC.

Interferon (IFN)-α (particularly IFN-α 2a) and interleukin (IL)-2 are cytokines routinely utilized clinically for the treatment of mccRCC. IFN-α is the largest isoform of interferon, with a broad spectrum of antiviral and immunomodulatory effects. Around 1972, IFN-α began to be employed in anticancer therapy (26). An early prospective randomized trial found that the total effective rate of IFN-α coupled with vincristine was 16.5%, higher than 2.5% of vincristine alone. And the median survival duration of patients who underwent combination therapy was 67.6 weeks, but that of patients who only received vincristine chemotherapy was 37.8 weeks (27). In 1994, IL-2 was approved for the treatment of mccRCC. In 1998, it was approved to treat metastatic melanoma (28). IL-2 has numerous biological functions, including activation of T cells, induction of cytotoxicity, boosting B cell proliferation, and secretion of antibodies, and other (29). All these evidences demonstrate that direct administration of exogenous cytokines can impact the development of tumors and modulation of the immune system is an effective cancer treatment.

Compared to chemotherapy, cytokine therapy does improve the responsiveness of patients with mccRCC. However, this therapy is associated with numerous adverse effects that severely affect the quality of patients’ survival. Adverse effects of IFN-α therapy include loss of appetite, fatigue, nausea, malaise, chills, and dry mouth (30, 31). Conventional doses of IL-2 have limited efficacy. Although higher doses increase the therapeutic capacity of IL-2, they can lead to adverse effects such as vascular leakage syndrome and other cytokine storms (32–34). Observational data on the efficacy of a group of patients treated with high doses of IL-2 for mccRCC showed that roughly 4% of patients died from adverse events related to IL-2 therapy (35). Numerous adverse events and low targeting are bottlenecks that are difficult to break through in cytokine therapy and are the main reasons for the gradual withdrawal of cytokines from the first-line medication ladder.

Notably, the cytokine era has developed a commonly used prognostic risk model for advanced/metastatic RCC, the Memorial Sloan Kettering Cancer Center (MSKCC) score. The score includes five risk factors, namely ① time from diagnosis to systemic therapy, ② physical status, ③ blood calcium, ④ hemoglobin, and ⑤ lactate dehydrogenase. According to the number of risk factors, patients could be divided into three groups: low, medium, and high risk, corresponding to a median OS of 30, 14, and 5 months, respectively (**Table 2**).

**Table 2:** Prognostic risk assessment criteria for advanced/metastatic RCC.

Risk factors	MSKCC criteria	IMDC criteria
①	Time interval between diagnosis and treatment <1 year	Time interval between diagnosis and treatment <1 year
②	Karnofsky physical status <80%	Karnofsky physical status <80%
③	Serum calcium > upper limit of normal index	Serum calcium > upper limit of normal index
④	Hemoglobin < lower limit of normal index	Hemoglobin < lower limit of normal index
⑤	Lactate dehydrogenase > 1.5 times the upper limit of the normal index	Neutrophils > upper limit of normal index
⑥		Platelet level > upper limit of normal index
Hazardous grouping		
Low-risk group	0 risk factor	0 risk factor
Medium risk group	1-2 risk factors	1-2 risk factors
High risk group	3-5 risk factors	3-6 risk factors

### 2.3 Targeted drug therapy

From about 2000 to 2010, targeting the vascular endothelial growth factor (VEGF) axis and mammalian target of rapamycin (mTOR) signaling pathway was the standard treatment to ccRCC (36). Von Hippel-Lindau (VHL) is the tumor suppressor of ccRCC and its inactivation blocks the degradation pathway of hypoxia inducible factor α (HIFα) (37). Highly expressed HIFα upregulates VEGF, thus promoting increased vascular permeability and angiogenesis, stimulating endothelial cell proliferation and migration, protecting endothelial cells from apoptosis, and promoting tumor invasion (38). Therefore, anti-angiogenesis has been used as a policy in targeting therapy. Currently, approaches to inhibit the VEGF pathway are broadly divided into two categories. The first type of strategy is to block the VEGF receptor (VEGFR) to block binding of VEGF to endothelial cells. The second type of strategy is to prevent the activation of VEGFR by human-derived monoclonal antibodies binding to circulating VEGF (39).

Tyrosine kinase inhibitor (TKI) is a multi-target receptor inhibitor. In ccRCC, TKI mainly inhibits VEGF signaling by targeting VEGFR-2 inhibition (38). TKI for mccRCC include sorafenib, sunitinib, pazopanib, axitinib, cabozantinib, lenvatinib, and tivozanib (40). Among them, the oral angiogenesis inhibitors sunitinib, pazopanib and cabozantinib are the first-line treatment options.

Sorafenib was the first TKI marketed for mccRCC. In phase II clinical study, sorafenib was not superior to IFN-α’s ability to prolong median progression-free survival (PFS). However, in terms of the proportion of patients with reduced tumor volume, sorafenib was higher than IFN-α (68.2% vs. 39.0%) (41). A 2007 clinical trial showed that objective response rate (ORR) in sunitinib-treated group was 31%, with a median PFS of 11 months, while the ORR was only 6% and a median PFS was 5 months in IFN-α-treated group (42). This trial demonstrated firstly that sunitinib was superior to IFN-α in treating mccRCC and led to replacement of cytokines by targeted drugs as the standard of care for mccRCC. Pazopanib was non-inferior to sunitinib to prolong PFS but had a better safety profile than sunitinib. For previously untreated patients, the pazopanib-treated group had an ORR of 30% and a median PFS of 11.1 months (43). Moreover, the incidence of adverse events such as hand-foot syndrome due to pazopanib treatment is lower than that of sunitinib (44). Axitinib inhibits intracellular VEGFR autophosphorylation with picomolar half maximal inhibitory concentration (IC_50_) values (45). Its IC_50_ for VEGFR1, VEGFR2, and VEGFR3 was significantly lower than sorafenib, sunitinib, or pazopanib. A randomized phase III trial divided 723 patients with advanced RCC into two groups. One group was treated with axitinib (5 mg twice daily), and one group received sorafenib (400 mg twice daily). The median OS in the axitinib-treated group was 20.1 months, the median PFS was 8.3 months, while the median OS in the sorafenib-treated group was 19.2 months, and the median PFS was 5.7 months (46, 47). The ability of axitinib to prolong OS was not significantly different from sorafenib. Nevertheless, its ability to prolong PFS was superior to sorafenib. This result establishes axitinib as a second-line treatment for mccRCC. Cabozantinib is an oral small molecule TKI that inhibits tumor vascular regeneration by inhibiting signaling pathways such as VEGFR and hepatocyte growth factor. A phase I trial used it to treat 25 patients with RCC who had failed standard therapy. Cabozantinib demonstrated preliminary antitumor activity and safety, with a median PFS of 12.9 months and a median OS of 15.0 months (48). Lenvatinib is a dual inhibitor of VEGFR and fibroblast growth factor receptor, which is generally used in combination with other drugs to treat RCC. The TIVO-3 trial compared tivozanib (1.5 mg once daily) with sorafenib (400 mg twice daily) in advanced RCC. The results showed that tivozanib did not differ from sorafenib’s ability to prolong OS. It was better than sorafenib in prolonging PFS (5.6 months vs. 3.9 months) as a third- or fourth-line treatment for mccRCC (49, 50).

Bevacizumab is the first recombinant humanized monoclonal antibody against VEGF. Patients have a median PFS of 10.2 months in the group treated with combination of bevacizumab and IFN-α, compared with 5.4 months in the group treated with placebo plus IFN-α (51, 52). Because bevacizumab, combined with IFN-α, significantly improved PFS in patients with mccRCC, it was approved by European Medicines Agency and US Food and Drug Administration (FDA) as a first-line agent for the treatment of advanced mccRCC around 2007. A phase III trial in 2022 showed that bevacizumab in combination with atezolizumab (an ICI) was not superior to sunitinib alone for the treatment of mccRCC (53). Currently, bevacizumab is not used in the treatment anymore.

mTOR inhibitors, which also inhibit angiogenesis, are an option for patients with advanced kidney cancer who are drug-resistant or failed to VEGF-targeted therapy. In ccRCC, the mTOR signaling pathway is hyperactivated to inhibit apoptosis and promote cancer cell proliferation and neovascularization (54). Compared to VEGF inhibitors, mTOR inhibitors are more potent in inhibiting cell proliferation and less potent in inhibiting neovascularization (55). In mccRCC, the commonly used mTOR inhibitors are temsirolimus and everolimus, both target mTOR complex 1 (56, 57). In a multicenter, phase 3 trial, 626 previously untreated patients were randomly assigned to the IFN-α treatment group, the temsirolimus treatment group, and the temsirolimus combined with IFN- α treatment group. The median PFS of patients in the three treatment groups were 3.1, 5.5, and 4.7 months, respectively. The median OS was 7.3, 10.9, and 8.4 months, respectively (58). Temsirolimus improved OS and PFS in patients with mccRCC compared to IFN-α, although the combination of temsirolimus with IFN-α was not superior to temsirolimus monotherapy. Unlike temsirolimus, administered intravenously, everolimus is an oral mTOR inhibitor. A randomized, double-blind phase 3 trial divided patients with mccRCC who received TKI therapy into two groups in a 2∶1 ratio: everolimus-treated and placebo groups. The median PFS in the two groups was 4.0 months and 1.9 months, respectively (59). Thus, everolimus became a recommended drug in clinical practice guidelines after the failure of first line VEGFR-TKI therapy (55).

Notably, the combination of lenvatinib with everolimus significantly improved patients’ median PFS (14.6 months vs. 5.5 months) and median OS (25.5 months vs. 15.4 months) compared to everolimus monotherapy. This combination regimen has been used in the second-line treatment of advanced RCC (60, 61). Similarly, a phase III trial compared cabozantinib with everolimus to treat advanced RCC. The trial divided 658 patients equally into a cabozantinib-treated group (60 mg once daily) and an everolimus-treated group (60 mg once daily). Cabozantinib showed an advantage in PFS (7.4 months vs. 3.8 months) and ORR (17% vs. 3%) compared to everolimus. Based on this, FDA approved cabozantinib for second-line treatment of metastatic RCC in 2016 (62). The Alliance A031203 CABOSUN trial divided 157 patients with metastatic RCC into a cabozantinib treatment arm (60 mg once daily), a sunitinib treatment arm (50 mg once daily; 4 weeks on, 2 weeks off). Cabozantinib had an even better ability to prolong median PFS (8.2 months vs. 5.6 months) and improve ORR (33% vs. 12%) compared to the standard first-line drug sunitinib (63, 64). Following the completion of the Phase II CABOSUN trial, FDA subsequently approved cabozantinib as first- and second-line therapy for mccRCC.

Although VEGF inhibitors and mTOR inhibitors can significantly improve disease control rates and prolong patient survival in ccRCC, they still cause a few adverse effects. TKI therapy frequently results in negative side effects such as hypertension, hand-foot syndrome, vomiting, and diarrhea (42). Adverse effects of bevacizumab are mainly manifested by its toxic effects on the gastrointestinal, cardiovascular, hematological, and urinary systems (65). Everolimus has fewer severe side effects, including common stomatitis, rash, and diarrhea (59). However, drug resistance and hyposensitivity are currently the most critical issues to be tackled with targeted therapies, not adverse events. Drug resistance in targeted drug therapy typically arises 6 to 11 months after treatment initiation and is caused by compensatory vascular proliferation due to hypoxic response (54, 66, 67). Tumor heterogeneity, the cause of hyposensitivity, is a huge problem to be conquered in the overall field of oncology treatment.

For the prognostic model of RCC, the International Metastatic Renal Cell Carcinoma Database Consortium (IMDC) criteria were established in the era of targeted therapy based on the MSKCC criteria. IMDC criteria include neutrophil and platelet levels as risk factors and exclude lactate dehydrogenase. Patients could be classified into three groups according to the risk factors: low, intermediate, and high risk, corresponding to a median OS of 35.3, 16.6, and 5.4 months, respectively (**Table 2**).

### 2.4 Immune checkpoint inhibitors therapy

ICI therapy is a new generation of immunotherapy designed to restore or improve the efficiency of the body’s immune system against tumors by abolishing the immune escape mechanism of tumors with ICI. Immune checkpoints are “brake pads” that negatively regulate T-cell function to avoid overactivation of the immune system. Programmed cell death 1 (PD-1), programmed cell death-ligand 1 (PD-L1), and cytotoxic T-lymphocyte associated protein 4 (CTLA-4) are all currently identified immune checkpoints (68). PD-1 and CTLA-4 are immune test sites distributed on the surface of T cells, and PD-L1 is the ligand for PD-1 that is highly expressed in tumor cells. Tumor cells could turn off the ability of T cells to kill tumor cells by overexpressing PD-L1. Cluster of differentiation (CD) 80 and CD86 expressed on antigen-presenting cells (APC) can bind to CTLA-4 on T cells and deliver signals that suppress immune responses. ICIs restore the aggressiveness of the immune system against tumor cells by blocking the binding process of the receptors mentioned above to ligands mentioned above. Nivolumab, pembrolizumab, atezolizumab, avelumab and ipilimumab are common ICIs used to treat mccRCC.

Nivolumab is a PD-1 inhibitor, and CheckMate-025 compared its efficacy with everolimus in advanced RCC (69). Subjects were patients who had previously received one or two kinds of anti-angiogenic therapies. Patients in the nivolumab-treated group had a median OS of 25 months, an ORR of 25%, and a median PFS of 4.6 months. Patients in the everolimus-treated group had a median OS of 19.6 months, an ORR of 5%, and a median PFS of 4.4 months. Although there was no significant difference in median PFS between the two groups, the ability of nivolumab to prolong OS and improve ORR was superior to everolimus. Pembrolizumab is also a common PD-1 inhibitor. In a single-arm phase II study, patients with advanced ccRCC were given 200 mg of pembrolizumab intravenously every three weeks. The ORR was 36.4%, and the disease control rate was 58.2% (70). A double-blind, phase 3 trial divided patients who had undergone nephrectomy into two groups. With 496 patients treated with pembrolizumab and 498 patients treated with placebo (71). Disease-free survival was significantly longer in the pembrolizumab-treated group compared to the placebo (disease-free survival at 24 months, 77.3% vs. 68.1%). It shows that pembrolizumab alone has shown excellent antitumor activity. Atezolizumab is a monoclonal antibody targeting the PD-L1 protein. A 2019 clinical trial assigned 915 previously untreated mccRCC patients to two groups: the atezolizumab combined with bevacizumab treatment group and the sunitinib treatment group. Treatment results showed that the median PFS of patients in the combined treatment group was 11.2 months and that in the sunitinib group was 7.7 months (72). Compared to sunitinib, the combination therapy prolonged PFS and showed a good safety profile but failed to gain OS benefit. Therefore, the combo atezolizumab-bevacizumab cannot be considered standard treatment compared to other with OS improvement. Avelumab is a PD-L1 antibody that can be used with axitinib to treat advanced RCC. In a phase III JAVELIN Renal 101 trial, 442 patients received the avelumab (10 mg/kg once every two weeks)-axitinib (5 mg twice daily) combination. Another 444 patients received sunitinib (50 mg once daily for the first 4 weeks of each 6-week cycle). Among the 560 PD-L1-positive patients, the median PFS (13.8 months vs. 7.2 months) and ORR (55.2% vs. 25.5%) were significantly higher in the combination treatment group than in the sunitinib treatment group (73). Ipilimumab, a monoclonal antibody to CTLA-4, was the first ICI approved for clinical use, initially for the treatment of metastatic melanoma (68). CheckMate-214 showed an ORR of 42% and a median PFS of 11.6 months in patients with advanced ccRCC treated with the combination of nivolumab and ipilimumab, significantly better than the 27% and 8.4 months, respectively, in the sunitinib-treated group (74). Moreover, the extended follow-up of this clinical trial showed that the combination therapy maintained a significant OS benefit compared to sunitinib (75). The superior OS and ORR make this immunotherapy combination a new standard of care option.

It is not difficult to discover that ICI therapy is always used in conjunction with other treatments to combat drug resistance. The pairwise or multiple combinations of the above-mentioned classical clinical treatments has become a research hotspot in the ccRCC area. For example, stereotactic ablative body radiotherapy (SABR) combined with ICI therapy, TKI and ICI therapy combined, the combination of two kinds of ICI therapy, and so on (75–77).

The RAPPORT trial evaluated the safety and efficacy of a short course of anti-PD-1 immunotherapy after SABR in patients with mccRCC. A single fraction of SABR to all metastatic sites followed by 200 mg pembrolizumab administered Q3W for eight cycles. The results showed excellent local control with an ORR of 63% and disease control of 83% (76). In the combination of TKI and ICI, the combos axitinib-pembrolizumab, lenvatinib-pembrolizumab, and cabozantinib-nivolumab are the standard of care for first-line setting. In a phase III trial, 861 patients were divided into axitinib (5 mg twice daily)-pembrolizumab (200 mg by IV every 3 weeks) treatment group or sunitinib treatment group (50 mg once daily for the first 4 weeks of each 6-week cycle). The median PFS of 15.1 months and ORR of 59.3% were significantly better in the combination treatment group than in the sunitinib treatment group at 11.1 months and 35.7% (78). In another phase III trial, 1069 patients with advanced RCC who had not received prior systemic therapy were randomized equally into three groups: the lenvatinib (20 mg once daily)-pembrolizumab (200 mg by IV every 3 weeks) treatment group, the lenvatinib (18 mg once daily)-everolimus (5 mg once daily) treatment group, or the sunitinib (50 mg once daily for the first 4 weeks of each 6-week cycle) treatment group. The median PFS in the three groups was 23.9 months, 14.7 months, and 9.2 months, respectively. Moreover, the OS was longer in the lenvatinib-pembrolizumab group than in the sunitinib group but not in the lenvatinib-everolimus group (79). This trial establishes lenvatinib-pembrolizumab as a first-line treatment. In addition, another trial investigated the efficacy and safety of cabozantinib (40 mg once daily)-nivolumab (240 mg every 2 weeks) compared to sunitinib (50 mg once daily for the first 4 weeks of each 6-week cycle) in advanced RCC. The one-year OS probability was 85.7%, and ORR was 55.7% in the cabozantinib-nivolumab treatment group, higher than the 75.6% and 27.1% in the sunitinib treatment group, respectively (80). A long-term follow-up showed a median OS of 37.7 months in the combination therapy group, slightly better than 34.3 months in the sunitinib group. Not only that, the ability of combination therapy to prolong median PFS was significantly better than sunitinib (16.6 months vs. 8.3 months). This follow-up further supports cabozantinib-nivolumab in the first-line treatment of advanced RCC (81). Nivolumab plus ipilimumab is a typical combination of two ICIs for treating advanced RCC. The CheckMate 214 trial divided the 1096 intent-to-treat population into a nivolumab-ipilimumab treatment arm or a sunitinib treatment arm. Extended follow-up showed that the combination therapy was superior to sunitinib in extending OS (median not reached vs. 37.9 months) and improving ORR (41% vs. 34%) in intent-to-treat patients (75).

While the survival rate of mccRCC patients is undoubtedly increased by the combination of classical clinical therapies, fully durable responses are rare currently (82). Additionally, combined therapies have limited effects on patients who are insensitive to anti-angiogenic therapies and have failed ICI treatment (83). Therefore, it is necessary to devote part of our efforts to the study of new therapeutic strategies.

## 3 Emerging potential therapeutic approaches

### 3.1 New strategies in clinical trials

Currently, three categories of emerging therapeutic strategies have entered clinical trials: ① new targeted drugs, ② HyperAcute Renal (HAR) immunotherapy, and ③ emerging drugs in combination with classic drugs (**Table 3**).

**Table 3:** Emerging therapeutic modalities in clinical research.

Type	Drug	Phase	Identifier
New targeted drug	Belzutifan	Phase II	NCT03401788
	Ciforadenant	Phase I/Ib	NCT02655822
	CDX-014	Phase I	NCT02837991
	Girentuximab	Phase I	NCT03556046
Emerging immunotherapy	HyperAcute renal immunotherapy	Phase I	NCT02035358
Emerging drug - Classical drug	Bempegaldesleukin - Nivolumab	Phase I/II	NCT02983045
	Telaglenastat - Everolimus	Phase II	NCT03163667
	Telaglenastat - Cabozantinib	Phase I	NCT02071862
	CBM588 - Nivolumab - Ipilimumab	Phase I	NCT03829111

**Figure 2:**
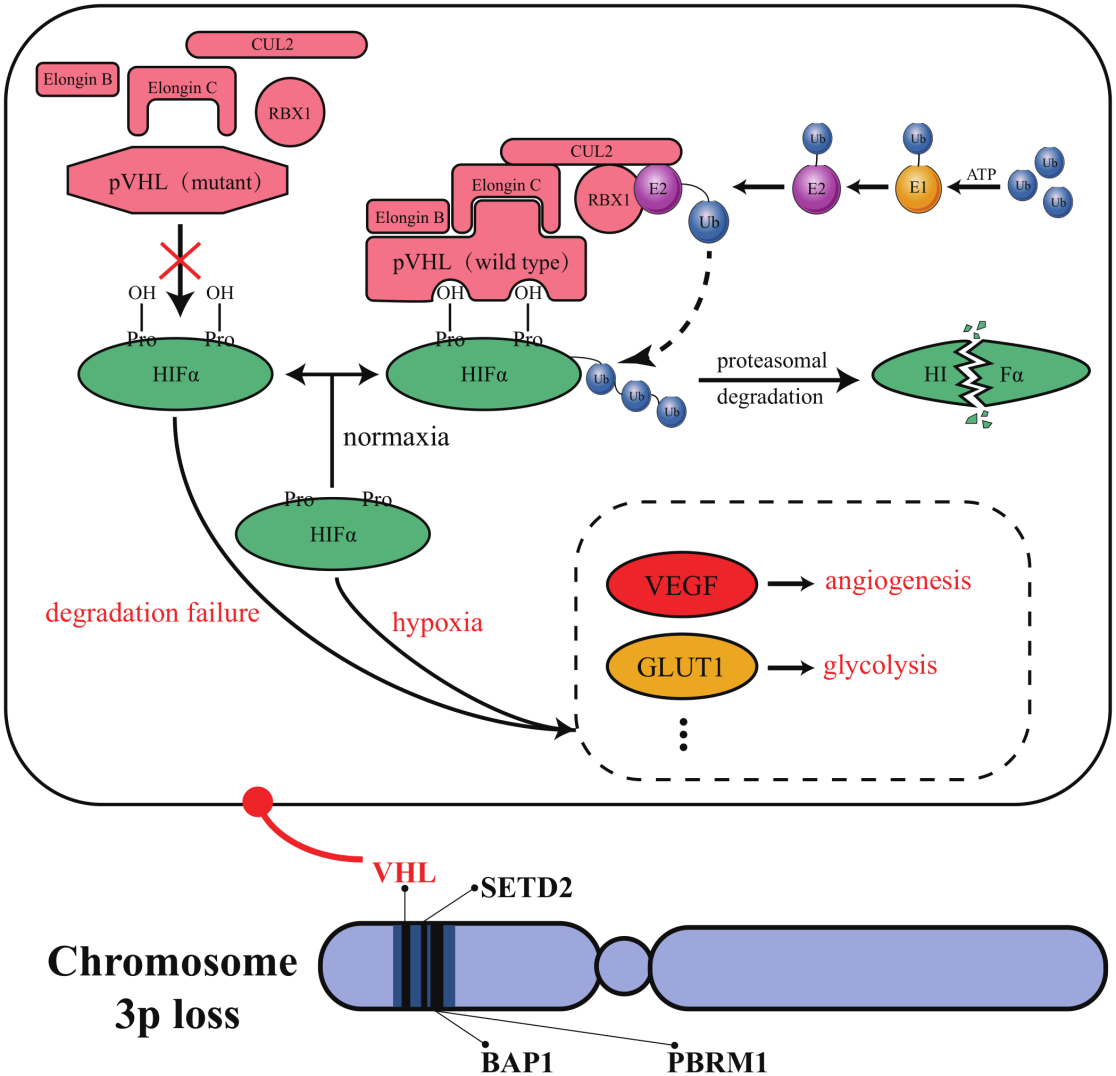
Molecular characteristics of ccRCC. VHL, PBRM1, BAP1, and SETD2 are all located in a region on the short arm of human chromosome 3. This region’s structural and functional deletion is known as “chromosome 3p loss”. Chromosome 3p loss has been confirmed as a common and carcinogenic driving event in ccRCC.

HIF2α inhibitors are undoubtedly the most promising emerging targeted drugs in recent years. As shown in **Figure 2**, the inactivation of VHL with blockage of the degradation pathway of HIFα is an essential molecular feature for the induction of ccRCC. VHL encodes pVHL, which connects with ElonginB and ElonginC through a short collinear region to form the VCB structure. VCB structure interacts with the amino-terminal of CUL2. At the same time, the carboxyl-terminal of CUL2 interacts with RBX1 of the Ring E3s to form a VBC (VHL-ElonginB/C-CUL2) complex (84). VBC complex is a common E3 ubiquitin ligase enzyme. The pVHL serves as the substrate recognition subunit of this complex and is responsible for targeting HIFα for degradation *via* the ubiquitin-proteasome pathway (85). HIFα is a hypoxia-inducible factor that includes HIF1α and HIF2α. Under normal conditions, it maintains the stability of the internal environment in response to tissue hypoxia. In ccRCC, VHL alteration leads to the inactivation of pVHL, which results in the inhibition of HIFα degradation (37). At this point, even if external oxygen is sufficient, the uninhibited HIF α begins to exert its biological effects. It enhances tumor cells’ growth and their resistance to treatment by regulating the expression of VEGF, glucose transporter-1 (GLUT1), and other downstream target genes to improve tissues’ angiogenesis and glycolysis ability (84). As described in section 2.3, most currently available targeted drugs target the downstream target VEGF, but Petolon Therapeutics has successfully developed HIF2α inhibitors in recent years. A phase I dose-escalation trial used the first-generation HIF2α inhibitor MK-3795 (previously known as PT2385) to treat previously treated patients with advanced ccRCC. Results showed that MK-3795 was well tolerated, with complete remission, partial remission, and disease stabilization rates of 2%, 12%, and 52%, respectively (86). In a mouse xenograft tumor model, the second-generation HIF2α inhibitor MK-6482 (also known as belzutifan, formerly PT2977) was approximately ten times more potent than MK-3795. Subsequently, belzutifan, which is more selective and has better pharmacological properties, was also entered into a phase I clinical trial. Results showed an ORR of 25% and a median PFS of 14.5 months in ccRCC patients treated with 120 mg of belzutifan once daily (87). Belzutifan showed good tolerability and preliminary antitumor activity. In late 2021, a Phase II single-arm trial of belzutifan was completed. Patients with RCC receiving 120 mg of belzutifan once daily had an ORR of 49%. Belzutifan demonstrated activity in treating VHL disease associated RCC (88). Furthermore, to guarantee a new treatment option for patients in the shortest possible time, FDA has granted belzutifan breakthrough drug status (89).

In addition, triggering adenosine 2A receptors (A2AR) are highly expressed in RCC. Adenosine binding to A2AR would mediate immunosuppression in the tumor microenvironment. Ciforadenant is a small molecule inhibitor that targets A2AR and has acted in various preclinical tumor models. In a phase I trial using ciforadenant to treat refractory RCC, the blocker showed a favorable safety profile and preliminary efficacy (90). Kidney injury molecule 1, also known as T cell immunoglobulin mucin-1 (TIM-1), is predominantly expressed on many immune cells and tumor cells. CDX-014, an antibody-coupled drug targeting TIM-1, has shown a controlled toxicity profile and preliminary activity in a phase I trial to treat advanced RCC (91). Carbonic anhydrase IX (CAIX) is a downstream target protein inhibited by VHL. Due to VHL mutations, 95% of ccRCC cells show overexpression of CAIX. Girentuximab, a chimeric monoclonal antibody effectively targets CAIX, has been reported to have good tolerability and tumor uptake, with efficacy to be further determined in subsequent experiments (92).

HAR immunotherapy is an emerging immunotherapy evaluated in many tumors. In RCC treatment, HAR consists of two allogeneic kidney cancer cell lines genetically modified to express alpha-1,3-galactosyltransferase (α (1,3)Gal). Because humans are intrinsically immune to α (1,3)Gal, HAR immunotherapy immunizes patients against metastatic RCC cells using naturally occurring xenograft and zoonotic infection barriers. The therapy is well tolerated in the kidney and has the potential as a candidate for metastatic RCC (93).

Consistent with multiple classical drug combination therapies, the fervor of new drug-older drug combinations in clinical research cannot be underestimated. Bempegaldesleukin is a prodrug drug for IL-2, modified to provide low toxicity, controlled and sustained activation of the IL-2 pathway, and stimulation of antitumor immune responses. The PIVOT-02 study demonstrated encouraging clinical activity and tolerability of bempegaldesleukin combined with nivolumab, warranting further study (94). Telaglenastat, an oral glutaminase inhibitor, is a crucial enzyme that supports abnormal tumor metabolism. Sixty-nine patients in the ENTRATA trial were divided into two groups in a 2:1 ratio and treated with either telaglenastat- everolimus or placebo-everolimus. The results showed that telaglenastat-everolimus treatment was well tolerated and better prolonged PFS in patients with advanced RCC compared to placebo-everolimus treatment (3.8 months vs. 1.9 months) (95). Similarly, telaglenastat in combination with cabozantinib showed good tolerability and clinical activity (96). CBM588 is a bifidogenic live bacterial product with the potential to enhance ICI response by modulating intestinal flora. In a phase I trial, 30 patients with RCC who had failed previous treatment were divided into two groups. One group was treated with CBM588 - nivolumab - ipilimumab, and the other group was treated with nivolumab - ipilimumab. PFS was significantly longer in the three-drug treatment group than in the two-drug combination group (12.7 months vs. 2.5 months). The effect of CBM588 on ICI treatment warrants further study (97).

### 3.2 New strategies in laboratory trials

#### 3.2.1 Potential molecular targets

The search for molecular targets with clinical application is not only crucial for early diagnosis and timely treatment of cancer but also can accelerate drug development and reduce ineffective experimental treatments (98). In recent years, the popularization of omics technology and bioinformatics technology has made it easier to find the potential targets of ccRCC. For example, secreted protein acidic and cysteine rich cysteine is a diagnostic urinary biomarker. Forkhead box M1, the epithelial-mesenchymal transition-related gene, can be used as a prognostic biomarker. The NADH dehydrogenase (ubiquinone) 1 alpha subcomplex, 4-like 2 can be used as an invasive marker (99–101). Applying bioinformatics technology to tumor studies has even shown explosive growth in recent years (102–105). Mining the cancer genome atlas and gene expression omnibus database has made it possible to obtain many differentially expressed molecular indicators that affect patient survival without much effort. However, to obtain reliable and valid biomarkers cannot rely on the mining of databases alone. The combination of computer technology and basic experiments may be a practical approach for future target exploration.

Polybromo 1 (PBRM1), BRCA1 associated protein 1 (BAP1), and SET domain containing 2 (SETD2) have also been reported as potential therapeutic targets for ccRCC. As tumor suppressor genes localized on human chromosome 3p21, PBRM1, BAP1, and SETD2 have a high mutation frequency in ccRCC (106). In ccRCC, PBRM1 is the second most mutation-prone gene after VHL (107). PBRM1 deletion is significantly related to high-grade tumors and poor patient prognosis, and its expression does not correlate with the mutational status of VHL (108). Loss of BAP1 occurs in approximately 15% of ccRCC patients (109). Interestingly, the mutations of BAP1 and PBRM1 are often mutually exclusive (110). A minority of ccRCC patients have tumors with simultaneous inactivation of BAP1 and PBRM1. These tumors in such patients exhibit rhabdoid features that predict aggressive tumor behavior (111). About 4-8% of ccRCC patients have SETD2 mutations, which inhibit autophagy and enhance cancer cell migration (112, 113). VHL, PBRM1, BAP1, and SETD2 are all located in a region on the short arm of human chromosome 3. This region’s structural and functional deletion is known as “chromosome 3p loss”. The occurrence of “chromosome 3p loss” has been confirmed as a common event and carcinogenic driving event in ccRCC (84). The expression of PBRM1 has been reported as a prognostic predictor in patients with mccRCC treated with TKI (114). Unfortunately, no new targeted agents against PBRM1, BAP1, or SETD2 have entered clinical trials.

#### 3.2.2 Tumor “slimming”

Obesity is one of the main risk factors for ccRCC, and the term “obesity” here refers to the “ body mass index (BMI) ≥30 kg/m^2^” defined by the WHO (115). Compared with people with normal BMI, people who are overweight (25 kg/m^2^ ≤ BMI <30 kg/m^2^) have a risk ratio of 1.28 for ccRCC, and obese people have a risk ratio of 1.82 (116). However, many analyses have pointed out that in ccRCC, the OS of obese patients is longer than that of non-obese patients (117). This phenomenon is known as the “obesity paradox”. A 2013 study showed that in ccRCC, patients in the normal BMI group had significantly higher fatty acid synthase (FASN) levels than those in the obese group (118). And the expression of FASN is related to the invasiveness and poor prognosis of cancer. This may provide an explanation for the obesity paradox. However, recently, it has been pointed out that although FASN is a significant predictor of poor prognosis in ccRCC patients, its level positively correlates with the proportion of visceral adipose tissue (VAT) (119). Thus, it seems that VAT is more closely related to ccRCC compared to lean body mass and subcutaneous adipose tissue. Since BMI cannot distinguish between adipose tissue and lean body mass, it may be inaccurate to simply define high BMI as a risk factor for ccRCC (118). Perhaps, stratifying obese patients according to the combined BMI and VAT% index before performing survival analysis could solve the puzzle of the obesity paradox.

The obesity paradox highlights the potential crosstalk between adipose tissue and tumors and leads us to focus on the role of lipids in ccRCC development and progression (120). Compared to normal renal tubular epithelial cells, ccRCC cells have a marked abnormality in lipid metabolism. Increased lipid synthesis and decreased β-oxidation level lead to massive deposition of lipids in the cytoplasm (121). This lipid deposition could stabilize cancer cell endoplasmic reticulum, relieve endoplasmic reticulum stress, and improve cell viability. So, whether regulating the lipid state of tumor cells can provide a new strategy for the treatment of ccRCC?

Several studies have shown that inducing the browning of white adipocytes is an effective strategy to reduce lipid deposition. White adipose tissue (WAT), brown adipose tissue (BAT), and beige adipose tissue are three types of fat present in the body. WAT is mainly energy storage, while BAT and beige play energy-consuming and heat-producing roles (122, 123). Browning is how WAT acquires BAT properties and promotes lipid consumption through heat production (124). Interestingly, ccRCC cells with aberrant lipid deposition can also undergo cellular browning, presenting thermogenic adipose characteristics, burning lipids, and “slimming” the cells. Studies have found that peroxisome proliferative activated receptor gamma coactivator 1 alpha (PGC1α) and phospholipase C like 1 (PLCL1) mediated cellular browning by regulating uncoupling protein 1 (UCP1) level could promote tumor “slimming” and inhibit tumor progression (125, 126). The concept of tumor “slimming” is defined as the phenomenon of lipolysis of large lipid droplets into tiny lipid droplets caused by cellular browning, and the size of tumor cells shrinks. In addition to the above molecules, nicotinamide nucleotide transhydrogenase (NNT) and carnitine palmitoyltransferase 1A (CPT1A) can also inhibit tumor proliferation, migration, and invasion through tumor “slimming” (127, 128).

Notably, in the view of tumor “slimming”, the browning of tumor tissue is an anti-cancer event, whereas a recent study has a different perspective. Wei et al. found a bi-directional communication between ccRCC tumor cells and adjacent PAT: ccRCC cells secrete parathyroid hormone-related protein, which promotes PAT browning through protein kinase A activation, while PAT-mediated thermogenesis leads to the release of excess lactate, thus promoting ccRCC growth, invasion, and metastasis (129). In addition, the adipocyte browning inhibitor H89 or KT5720 can block this bi-directional communication and enhance the anti-tumor effect of sunitinib. So, the exact role of browning is still confusing in ccRCC.

We speculate that browning browning may play a double-edged role in the development and progression of ccRCC. When browning occurs in ccRCC cells, it is cancer-suppressing, while browning in PAT is cancer-promoting. Therefore, tumor “slimming” is still a potential effective strategy to treat ccRCC. However, how to make the browning occur only in ccRCC cells, that is, how to make the “slimming” oriented, will be a problem that must be solved in this strategy in further.

#### 3.2.3 Identification and killing of renal cancer stem cells

Available evidence suggests that the initial carcinogenic mutations in solid tumors occur in cancer stem cells (CSCs) (130), a small group of cells with stem cell-like characteristics in tumor tissues, which have strong tumorigenicity, radiotherapy and chemotherapy resistance, and multiple drug resistance, which is an actual cause of tumor metastasis and recurrence (131). Therefore, CSCs should be considered as a priority target in cancer treatment. Unfortunately, CSCs are inherently resistant to some existing standard treatments. The extremely low proportion of CSCs in cancer tissues also brings great difficulties for its identification.

Now, the existence, localization, and markers of renal CSCs are still controversial. Some studies found that CD24, integrin beta 1, and prominin 1 can be used as renal CSCs markers (132). However, researchers from Birchmeier’s laboratory stated that CD24 and integrin beta 1 are expressed in most ccRCC cells and cannot be used as criteria to identify renal CSCs. Not only that, they also found that renal CSCs were positive for C-X-C motif chemokine receptor 4 (CXCR4), mesenchymal-epithelial transition factor (MET) and homing cell adhesion molecule (CD44), which accounted for an average of 2.2% of the total tumor cells (133). WNT, NOTCH signaling is significantly activated in CXCR4^+^MET^+^CD44^+^ triple-positive cells. The WNT inhibitor ICG-001 dramatically reduced ccRCC xenograft tumor volume *in vivo*, which showed that blockade of WNT signaling might be an effective treating strategy for ccRCC.

Shinya Tanaka’s team argued that it was impossible to identify CSCs clinically in individual patients. To establish a rapid detection method for CSCs, they established a method to reprogram cancer cells into CSCs. The team selected six human cancer cell lines for the experiment: KMG4 (brain cancer), HeLa (cervical cancer), A549 (lung cancer), WiDr (colon cancer), J82 (bladder cancer), and Fuji (synovial sarcoma). When these cancer cells were placed on a double-network hydrogel (DN gel) composed of PAMPS and PDMAAm, they were reprogrammed to CSC (134). If the DN gel induction of CSC can be applied to ccRCC, then it will be helpful to identify new markers of renal CSCs. In addition, the use of DN gel in combination with biopsy specimens from ccRCC patients would allow for early cancer diagnosis. This technology also facilitates high-throughput screening of CSC killing reagents.

#### 3.2.4 Strengthening the intrinsic immune system

Currently, strengthening the intrinsic immune system can be divided into “turning off the brakes” and “stepping on the gas”. “Turning off the brakes” means lifting the tumor suppression of the immune system, such as ICI therapy. “Stepping on the gas” is to activate the immune system through highly effective and low-toxic immunostimulatory drugs, which is a potential treatment based on cytokine therapy.

In terms of “turning off the brakes”, only a few patients can gain long-term survival benefits from ICIs treatment. One of the possible reasons for this phenomenon is the presence of many immunosuppressive cells in the tumor microenvironment. The large number of immunosuppressive cells recruited by tumors also play a vital role in establishing a pre-metastatic niche (135). These immunosuppressive cells include myeloid-derived suppressor cells, tumor-associated macrophages, regulatory T cells (Treg), regulatory dendritic cells, and others. Existing studies have shown that tumors can change the phenotype and function of normal immune cells from a potentially tumor-reactive state to a tumor-promoting state (136). This effect is closely related to the failure of cancer immunotherapy. Gang Xue et al. found that Ecto-5’-Nucleotidase (CD73), which is highly expressed by most types of immunosuppressive cells and some tumor cells, may be an essential target protein for the clearance of immunosuppressive cells (137). Moreover, they designed an IR-700 dye-conjugated anti-CD73 monoclonal antibody (αCD73-DYE). Under exposure to near-infrared irradiation (690 nm), αCD73-DYE specifically binds and kills CD73^+^ cells without damaging neighboring cells. This simultaneous depletion of tumor and immunosuppressive cells significantly ameliorated ICIs drug resistance.

In terms of “stepping on the gas”, research teams have developed a strong interest in modifying cytokines and T cells in order to improve therapeutic targeting. As we mentioned earlier: IL-2 is one of the central cytokines for inducing cellular immunity, but the resulted adverse effects limit its application. Researchers have designed a precursor drug for IL-12 (pro-IL-12) to address this problem. Before reaching the tumor, pro-IL-12 is inactive; it exerts anti-tumor effect only when it reaches the tumor. Moreover, pro-IL-12 treatment can have a synergistic effect with TKI therapy (138). Meanwhile, K Dane Wittrup’s team noted the anti-tumor effect of IL-12. They found that intratumoral injection of alum-tethered IL-12 induced safe and effective anti-cancer immunity (139). These alum aggregates were able to form a physical depot at the injection site that is persistent over several weeks, which allowed the IL-12 to bound to it to remain in the tumor for more than a week. This approach has produced robust local, systemic anti-tumor responses in multiple preclinical models and caused negligible systemic toxicity after administration. This research team has applied for a patent for this therapeutic technique and will soon be conducting clinical trials. In addition, T cell modification is also an auspicious tool for anti-cancer therapy. For example, a tumor chimeric antigen receptor is assembled on T cells, known as chimeric antigen receptor T-cell (CAR-T) immunotherapy. However, CAR-T therapies suffer from short cell lifespan and low antigen density ineffectiveness. Maria Themeli et al. developed dual receptor T cell therapy to address this problem (140). That is, another receptor is introduced into CAR-T: the chimeric co-stimulatory receptor (CCR). Dual receptor T cells double the anti-cancer capacity of T cells, enhancing their sensitivity to low antigen density and promoting their persistence. However, the response rate of dual receptor T-cell therapy in solid tumors may not be as good as in hematologic cancers. Perhaps, with the alum tethered method of K Dane Wittrup’s team, the modified dual receptor T cells can exert a safe and effective anti-tumor response in solid tumors like ccRCC.

## 4 Conclusion and prospect

This article reviews classical clinical treatments and emerging potential therapeutic approaches for ccRCC. Based on the molecular mechanisms of tumorigenesis and progression, a series of treatments for ccRCC have been explored. Among them, surgery, tumor ablation, cytokine therapy, anti-angiogenic therapy, and ICIs therapy are relatively mature treatments that have been applied in the clinic. In order to achieve better survival benefits for patients, current research focuses on two or more combinations of these mature therapies. However, exploring new therapeutic approaches is necessary for patients (especially mccRCC patients) who are insensitive to mature treatments or fail to treat them. This article lists several immature but promising therapeutic approaches for ccRCC.

**Figure 3:**
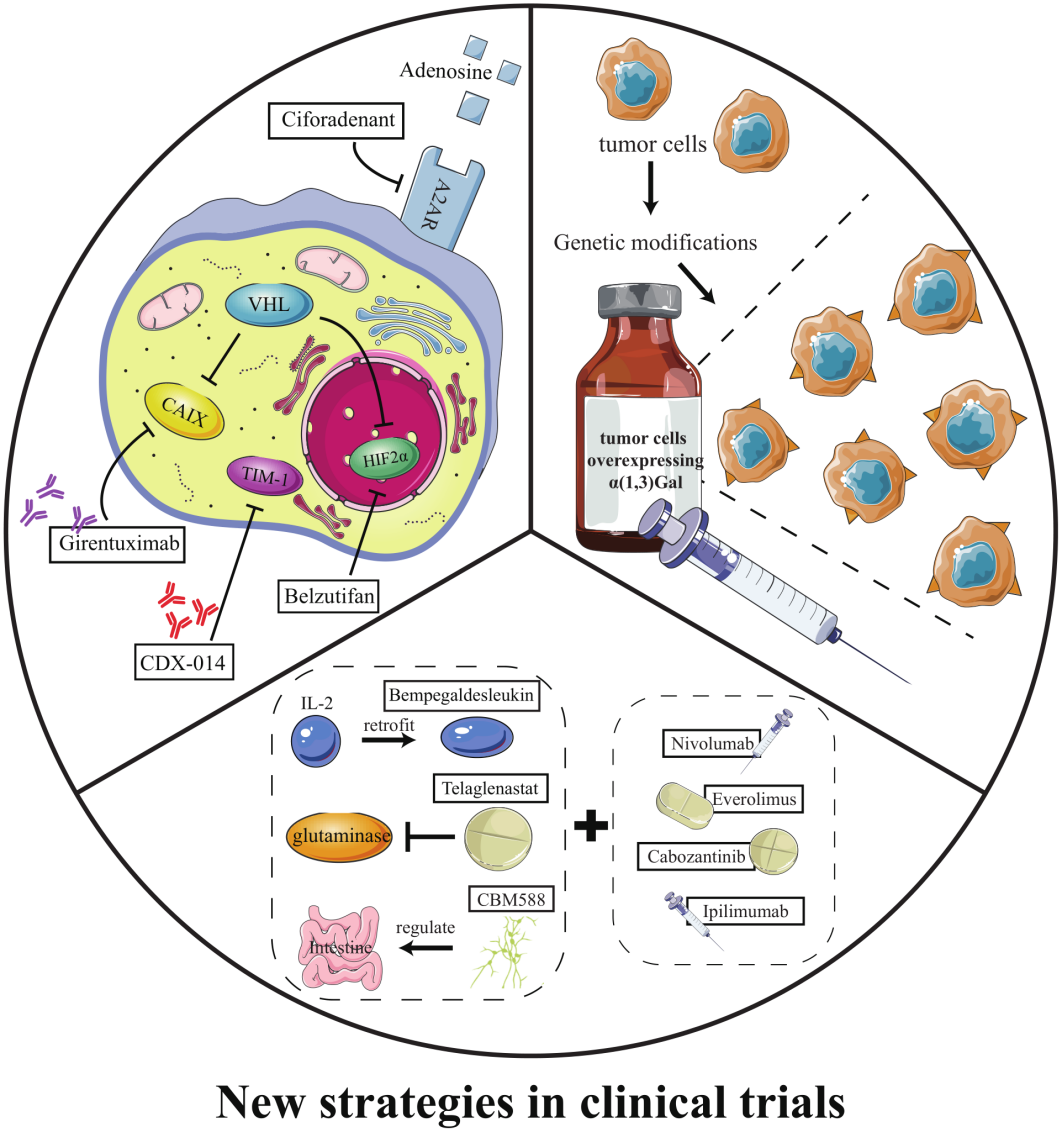
New strategies in clinical trials. (i) new targeted drugs; (ii) HAR immunotherapy; (iii) emerging drugs in combination with classic drugs.

**Figure 4:**
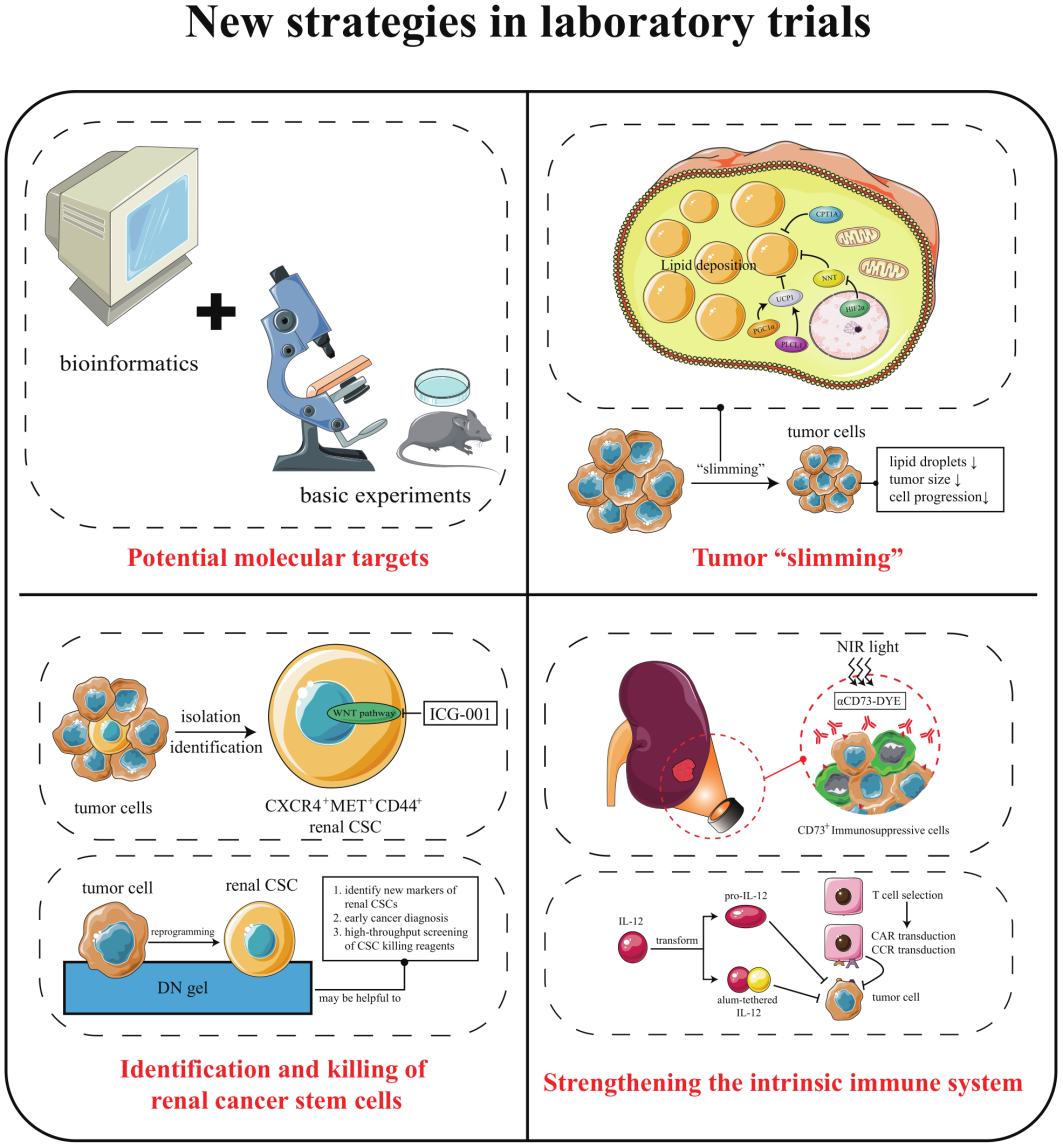
New strategies in laboratory trials. (i) combining computer technology with basic experiments to uncover new molecular targets of ccRCC; (ii) eliminating abnormal lipid deposition in ccRCC cells, thereby inhibiting tumorigenesis and progression; (iii) improving the ability to identify and kill renal CSCs; (iv) continuing to focus on the immune system to lift immunosuppression and “stepping on the gas”.

As shown in **Figure 3**, developing new targeted drugs, modifying immune substances, and modulation in the tumor microenvironment are essential aspects of clinical trials. Among them, belzutifan, which targets the classical target HIF2α, is the most promising drug. Laboratory trials, on the other hand, offer more options for clinical trials (**Figure 4**). ① Using omics and bioinformatics technologies to mine new biomarkers of ccRCC can provide new molecular targets for drug development; ② Focusing on the metabolic reprogramming of ccRCC, eliminating abnormal lipid deposition in cancer cells to inhibit tumorigenesis and progression; ③ Identifying and killing kidney CSCs to inhibit ccRCC metastasis and recurrence; ④ Removing immunosuppressive cells and modifying cytokines or T cells to strengthen the anti-cancer ability of the internal immune system.

The above treatments have shown remarkable results in laboratory or clinical trials. At the same time, the available studies have revealed some noteworthy issues. For example, the potential crosstalk between multiple adipokines and ccRCC is too unclear to make tumor “slimming” directional. There is no scientific means to precisely define patients who can benefit from a particular therapy, which may lead to over-medication or ineffective medical treatment. However, we believe that with the deeper integration of computer technology and basic experiments, the mechanisms underlying the development of ccRCC will become more apparent. Moreover, with more and more medical public databases are established, more biomarkers for diagnosis and prognosis will be excavated. In short, advances in technology will lead to a greater diversity of treatment options for ccRCC. Then, selecting and formulating the best treatment plan for the target population will become an important issue to achieve precision medicine in the future.

## Author contributions

JY wrote the manuscript. KW and ZY critically reviewed and edited the manuscript. All authors contributed to the article and approved the submitted version.

## Glossary

**Table:**

APC	antigen-presenting cells
A2AR	triggering adenosine 2A receptors
BMI	body mass index
BAT	brown adipose tissue
BAP1	BRCA1 associated protein 1
ccRCC	clear cell renal cell carcinoma
CN	cytoreductive nephrectomy
CA	cryoablation
CTLA-4	cytotoxic T-lymphocyte associated protein 4
CPT1A	carnitine palmitoyltransferase 1A
CSCs	cancer stem cells
CXCR4	C-X-C motif chemokine receptor 4
CD44	homing cell adhesion molecule
CD73	Ecto-5’-Nucleotidase
CAR-T	chimeric antigen receptor T-cell
CCR	chimeric co-stimulatory receptor
CD	cluster of differentiation
CAIX	carbonic anhydrase IX
DN gel	double-network hydrogel
FDA	Food and Drug Administration
FASN	fatty acid synthase
GLUT1	glucose transporter-1
HIFα	hypoxia inducible factor α
HAR	HyperAcute Renal
ICI	immune checkpoint inhibitor
IFN	interferon
IL	interleukin
IC_50_	half maximal inhibitory concentration
IMDC	International Metastatic Renal Cell Carcinoma Database Consortium
mccRCC	metastatic clear cell renal cell carcinoma
MWA	microwave ablation
mTOR	mammalian target of rapamycin
MET	mesenchymal-epithelial transition factor
MSKCC	Memorial Sloan Kettering Cancer Center
NSS	nephron-sparing surgery
NNT	nicotinamide nucleotide transhydrogenase
OS	overall survival
ORR	objective response rate
PAT	perinephric adipose tissue
PFS	progression-free survival
PD-1	programmed cell death 1
PD-L1	programmed cell death-ligand 1
PGC1α	peroxisome proliferative activated receptor gamma coactivator 1 alpha
PLCL1	phospholipase C like 1
pro-IL-12	precursor drug for interleukin-12
PBRM1	polybromo 1
RCC	renal cell carcinoma
RN	radical nephrectomy
RFA	radiofrequency ablation
SABR	stereotactic ablative body radiotherapy
SETD2	SET domain containing 2
TKI	tyrosine-kinase inhibitor
Treg	regulatory T cells
TIM-1	T cell immunoglobulin mucin-1
UCP1	uncoupling protein 1
VEGF	vascular endothelial growth factor
VHL	Von Hippel-Lindau
VEGFR	vascular endothelial growth factor receptor
VAT	visceral adipose tissue
WAT	white adipose tissue
αCD73-DYE	anti-CD73 monoclonal antibody
α (1,3)Gal	alpha-1,3-galactosyltransferase
